# Performance evaluation of lossy quality compression algorithms for RNA-seq data

**DOI:** 10.1186/s12859-020-03658-4

**Published:** 2020-07-20

**Authors:** Rongshan Yu, Wenxian Yang, Shun Wang

**Affiliations:** 1 Department of Computer Science, Xiamen University, Xiamen, 316005 China; 2 Aginome Scientific, Xiamen, 316005 China

**Keywords:** RNA-seq, Lossy compression, Base quality

## Abstract

**Background:**

Recent advancements in high-throughput sequencing technologies have generated an unprecedented amount of genomic data that must be stored, processed, and transmitted over the network for sharing. Lossy genomic data compression, especially of the base quality values of sequencing data, is emerging as an efficient way to handle this challenge due to its superior compression performance compared to lossless compression methods. Many lossy compression algorithms have been developed for and evaluated using DNA sequencing data. However, whether these algorithms can be used on RNA sequencing (RNA-seq) data remains unclear.

**Results:**

In this study, we evaluated the impacts of lossy quality value compression on common RNA-seq data analysis pipelines including expression quantification, transcriptome assembly, and short variants detection using RNA-seq data from different species and sequencing platforms. Our study shows that lossy quality value compression could effectively improve RNA-seq data compression. In some cases, lossy algorithms achieved up to 1.2-3 times further reduction on the overall RNA-seq data size compared to existing lossless algorithms. However, lossy quality value compression could affect the results of some RNA-seq data processing pipelines, and hence its impacts to RNA-seq studies cannot be ignored in some cases. Pipelines using HISAT2 for alignment were most significantly affected by lossy quality value compression, while the effects of lossy compression on pipelines that do not depend on quality values, e.g., STAR-based expression quantification and transcriptome assembly pipelines, were not observed. Moreover, regardless of using either STAR or HISAT2 as the aligner, variant detection results were affected by lossy quality value compression, albeit to a lesser extent when STAR-based pipeline was used. Our results also show that the impacts of lossy quality value compression depend on the compression algorithms being used and the compression levels if the algorithm supports setting of multiple compression levels.

**Conclusions:**

Lossy quality value compression can be incorporated into existing RNA-seq analysis pipelines to alleviate the data storage and transmission burdens. However, care should be taken on the selection of compression tools and levels based on the requirements of the downstream analysis pipelines to avoid introducing undesirable adverse effects on the analysis results.

## Background

In recent years, high-throughput genome sequencing has become an essential tool in biomedical and medical research with a wide range of applications in basic biomedical research, clinical diagnostic, drug discovery, forensic medicine, etc. The fast growing applications of genomic data have produced a large amount of sequencing data, for which efficient and effective data compression technologies are desired to cope with the corresponding growth in data storage and transmission costs.

To address such a need, genomic data compression algorithms have been proposed in the literature [1]. Many of these algorithms use a reference-based method where instead of the raw sequence data, alignments and mismatches of the reads against a standard reference sequence are coded [2, 3]. By using reference-based approaches, the nucleotide sequences in the reads can be heavily compressed. As a result, the base quality values, which carry information about the likelihood of each base call being in error, become the major component in the compressed sequencing data due to their high entropy. It was reported that base quality values can take up to 80% of a losslessly compressed file in size [4].

To further improve the data size reduction rate of genomic data compression, lossy compression algorithms of quality values [5, 6] have been proposed, where quality values were further quantized to coarse granularity to reduce the entropy. For lossy genomic data compression, it is expected that it could bring sizable gain in terms of file size reduction over lossless compression such that the storage burden can be significantly alleviated. Moreover, it is expected that the efficiency, accuracy, and reliability of downstream analysis should not be notably affected when lossy compression is adopted. To this end, many algorithms use highly customized quantization schemes that incorporate biological information from the data to maximize compression ratio without introducing significant impacts to downstream analysis [7–9]. As a result, artifacts introduced by lossy quality value compression to the analysis results may be both data-dependent and processing pipeline-dependent, which have to be carefully evaluated in order to select the best compression tool that achieves the desired trade-off between compression and data quality for the target application.

As genomic data compression algorithms were designed for standard sequencing data formats such as FASTQ and SAM, they can be used to compress both DNA and RNA sequencing data directly. However, most existing genomic data compression algorithms were designed for and evaluated on DNA sequencing data from whole genome sequencing (WGS), whole exome sequencing (WES) or targeted amplicon sequencing, etc. [4]. The impacts of lossy quality value compression on RNA-seq data analysis have thus far not been systematically evaluated in the literature, despite the increasing popularity of RNA-seq studies in both basic biomedical research and clinical applications, and the equivalently huge amount of sequencing data generated from RNA-seq studies [10–12].

In this paper, we provide a systematic evaluation on the impacts of lossy quality compression on common RNA-seq data analysis pipelines including gene expression quantification [13], transcript alternative splicing [14, 15] and short variants detection [16, 17]. We chose four datasets from four different species and sequencing platforms in our evaluation. We split each dataset into three equal parts, and treated the split parts as technical replicates of one experiment under the same conditions of the same sample. This enables us to evaluate the significance level of the impact from compression using statistical methods. Finally, analysis results from uncompressed data were used as groundtruth in our studies to avoid possible confounding factors other than lossy quality value compression when other external groundtruth results were used.

## Results

### Compression performance

**Fig. 1:**
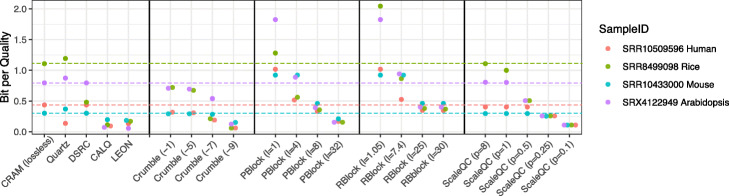
Compression performance (BPQ) of different quality value compression algorithms. Here, average BPQ of different compression algorithms on three technical replicates of each dataset are shown. Vertical lines indicate BPQ of lossless quality value compression by CRAM

The compression ratio of quality values varied depending on the datasets and the lossy compression algorithms and their corresponding coding parameters being used (Fig. 1). As a baseline, lossless compression using CRAM achieved a BPQ of 1.11 on SRR8499098 (the rice dataset), 0.3 for SRR1043300 (the mouse dataset), 0.436 for SRR10509596 (the human cell line dataset), and 0.795 for SRX4122949 (the arabidopsis dataset). Strikingly, not all lossy algorithms achieved better compression performance compared to CRAM. For methods with a fixed compression level, Quartz achieved higher compression ratio than CRAM only on SRR10509596 and Illumina binning (DSRC) only on SRR8499098. On the other hand, Crumble, LEON and CALQ achieved better results than CRAM on all four datasets. For methods where the compression level can be adjusted by input parameters, P-block achieved better performance compared to CRAM on all four datasets at its highest compression level, but not at other compression levels. R-block did not reach the lossless performance baseline on SRR1043300 even at its highest compression level in our test. ScaleQC is a scalable compression solution that enables both lossless and lossy compression. At lossless compression level (-p 8), it showed a comparable compression performance to CRAM. As ScaleQC supports bit-stream level scalability, higher compression ratio can be achieved by further truncating the losslessly compressed bit-stream to lower data rates.

**Fig. 2:**
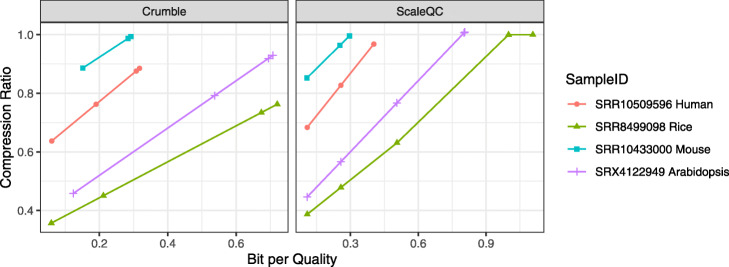
Overall compression ratio of Crumble and ScaleQC with respect to file sizes of original CRAM files at different bit-per-quality values for different sequencing files. As quality values occupy a significant portion of total sequencing data file, the overall compression ratio is highly related to the BPQ. Lossless compression was achieved by ScaleQC at its highest BPQ

As quality values occupy a significant portion of the total sequencing data file, the overall compressed file sizes are highly related to the sizes of quality values after compression (Fig. 2). Overall, at the lowest BPQ values, Crumble and ScaleQC achieved further file size reduction and the resulting file size ranges from 0.38 to 0.83 times of the size of the CRAM lossless baseline. The highest file size reduction with regard to the lossless baseline was achieved by Crumble on the rice dataset (SRR8499098) sequenced by HiSeq2500. On the other hand, the mouse dataset (SRR10433000) sequenced by NovaSeq 6000 was the most challenging dataset for lossy value compression, for which the highest file size reduction with regard to the lossless baseline was achieved by ScaleQC at its highest compression level. Note that this dataset has a lower BPQ when losslessly compressed by CRAM compared to other datasets (Fig. 1). Interestingly, ScaleQC also achieved slightly higher overall compression ratio compared to CRAM on the human cell line dataset (SRR10509596) at its highest BPQ setting, where it achieved lossless quality value compression.

### Alignment to reference genome

STAR [18] and HISAT2 [19] are among the most popular aligners used in RNA-seq analysis pipelines [1]. As the STAR method does not utilize base quality values for alignment, lossy quality value compression does not affect the alignment results and the SAM files it produces do not differ with or without lossy compression except for the base quality values themselves. On the other hand, HISAT2 uses base quality values to calculate the marginal penalty incurred by a mismatch at specific read positions, and the alignment results differ if base quality values are lossy compressed.

**Fig. 3:**
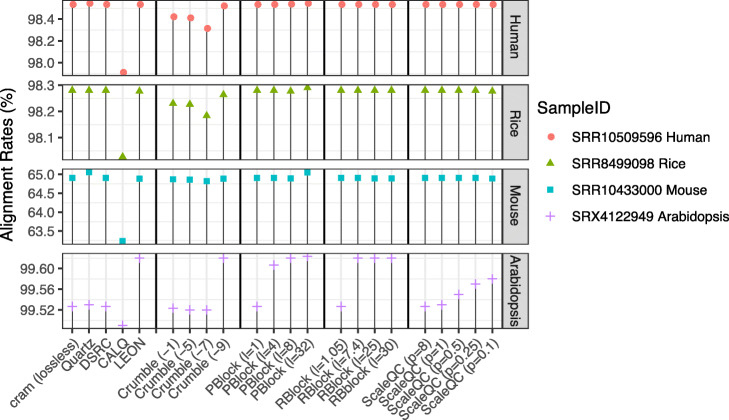
Average alignment rates of HISAT2 on three replicates of different datasets with original quality values and quality values lossy compressed by different algorithms

We compared the alignment rates of HISAT2 with original and lossy compressed quality values (Fig. 3). Noticeably, the mouse dataset (SRR1043300) has relatively lower mapping rate (< 65%) compared to the other three datasets regardless whether the quality values were lossy compressed or not. For all four datasets, the impact of different lossy compression on the alignment results of HISAT2 is very small regardless of the compression ratio except for CALQ, of which the alignment rates were around 0.3% to 2% lower than those of other algorithms. Although the difference is relatively small, such degradation could be a potential origin of non-negligible adverse effects on the downstream analysis for HISAT2-based pipelines. Crumble also introduced noticeable reduction in the alignment rates on the human cell line dataset (SRR10509596) and the rice dataset (SRR8499098), but to a lesser extent. Interestingly, Crumble introduced less degradation to alignment rate at its highest compression level (-9), suggesting that it is possible to further optimize its performance on RNA-seq data analysis at moderate compression levels.

### Gene expression quantification

The methods for gene expression quantification include alignment-based methods which align the reads to either a reference genome (e.g., featureCounts, HTSeq-count [20]) or a reference transcriptome (e.g., Salmon, RSEM [21]), and alignment-free algorithms which quantify expression levels by directly counting the *k*-mers (e.g., Kallisto [22] and Salmon). For alignment-based methods, since gene expression quantification methods rely on alignment information rather than base quality values, lossy quality value compression will affect the quantification results only when the alignment results are affected. In addition, quality values are not utilized in *k*-mer based alignment-free algorithms. Therefore, we only considered HISAT2 + featureCounts in our evaluation.

**Fig. 4:**
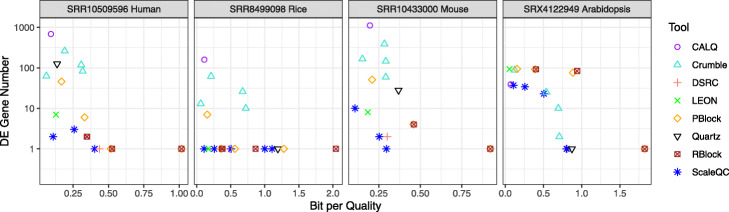
Number of DE genes due to lossy compression with respect when different compression methods and coding parameters were used in HISAT2 + featureCounts. *p*-values were evaluated using one-way ANOVA on three technical replicates for each dataset. *p*-values smaller than 0.05 were considered as significant

**Fig. 5:**
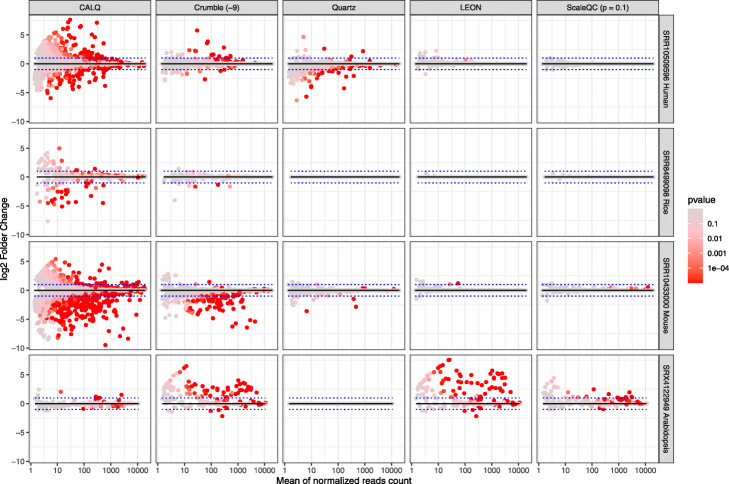
MA plot showing DE genes due to lossy compression for ScaleQC (-p 0.1), LEON, CALQ, Quartz, and Crumble. Dots color showing *p*-value calculated by DeSeq, genes with lower *p*-values were dyed with red, indicating significant expression differences due to lossy compression

For each dataset, we evaluated the number of genes that were identified as differentially expressed (DE) after lossy quality value compression on the quantification results from three technical replicates. Here, we analysed the variances of read counts between and within groups of quantification results from original and lossy compressed quality values for all the genes from each dataset, where within-group variances reflect randomized effects due to sampling and pipeline processing, and between-group variances represent the impacts of lossy compression. Significance were evaluated using one-way ANOVA and genes with *p*-value smaller than 0.05 were considered as DE genes. Reads counts were used in our benchmarking without further normalization since the total read counts were fixed in each split samples. As shown in Fig. 4, in general, higher compression ratios led to larger number of DE genes. However, the influence of lossy quality value compression on quantification results depends on the algorithms being used. Noticeably, ScaleQC and LEON were able to compress the quality values with minimal influences on gene expression quantification at high compression ratios, in particular in the rice dataset (SRR8499098). On the other hand, CALQ, Quartz and Crumble created more DE genes compared to other methods. We further compared DE genes of ScaleQC (-p 0.1), LEON, CALQ, Quartz and Crumble (-9) using mean-average (MA) plots (Fig. 5), demonstrating the adverse effect of lossy compression by CALQ, Crumble and Quartz on gene expression quantification when HISAT2 + featureCounts were used.

### Alignment-based transcriptome assembly

We included two alignment-based transcriptome assembly algorithms, namely, StringTie and Scallop, in our evaluation. Since both tools use only alignment information in their transcriptome assembly process, we used HISAT2 with these two tools to study the impact of lossy compression on alignment-based transcriptome assembly. In our test, we compared the assembled transcriptomes using GffCompare [23] against the constructed isoforms from data with original quality values as groundtruth. Performance was evaluated using precision, sensitivity and F1 score defined as:

Sensitivity = TP / (TP + FN),

Precision = TP / (TP + FP),

F1 score = 2 × Sensitivity × Precision / (Sensitivity + Precision).

Here, TP (true positive) is the number of transcripts in query in agreement with the corresponding reference, FP (false positive) is the number of transcripts in query that are not present in the reference, and FN (false negative) is the number of transcripts that are present in the reference but not in the query data.

**Fig. 6:**
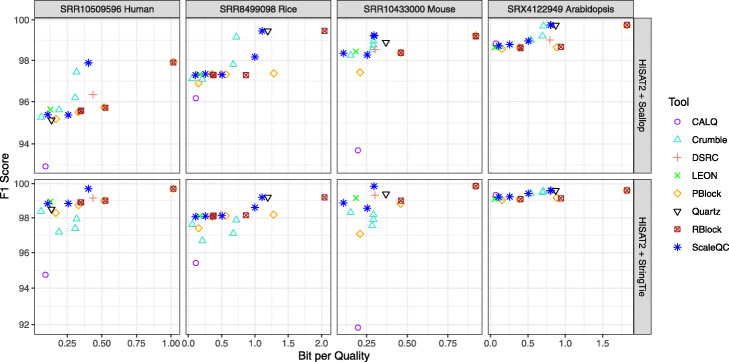
F1 scores of the reconstructed isoforms from lossy compressed RNA-seq data with respect to those from uncompressed data. The shown F1 scores were averaged on three replicates of each dataset

Based on the results (Fig. 6 and Supplementary Figure), lossy compression indeed affected the isoform reconstruction results when HISAT2 was used as aligner. In particular, the impacts were larger when Scallop was used compared to StringTie. Among all the lossy compression algorithms, the impacts of lossy compression on the consistency of reconstructed isoform were similar regardless of compression tools and coding parameters, except for CALQ which resulted in notably greater difference in reconstructed isoforms compared to other tools.

### Short variants detection

**Fig. 7:**
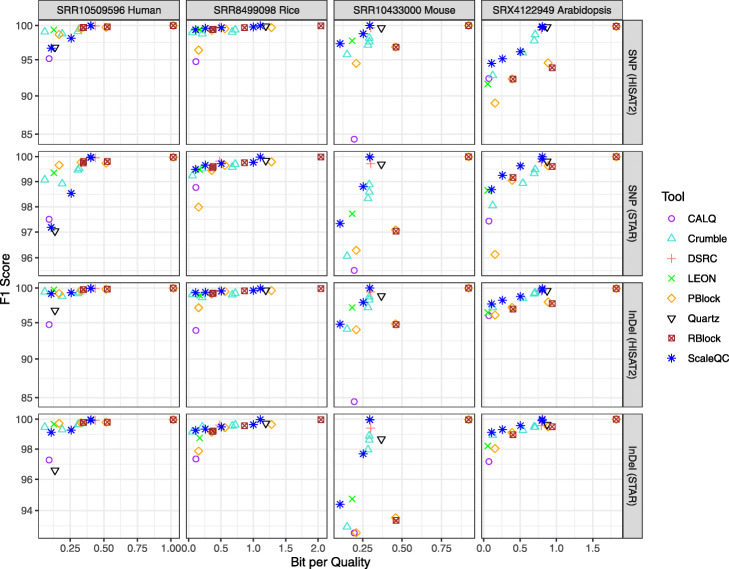
F1 scores of variant calling results on lossy compressed RNA-seq data using those from original RNA-seq data as groundtruth reference. The shown F1 scores were averaged on three replicates of each dataset

In contrast to previously evaluated RNA-seq analysis tools, short variant discovery tools, such as the GATK HC pipeline, rely heavily on quality values in the variant discovery process. For this reason, we included both aligners, STAR and HISAT2, in our evaluation. Sensitivity and precision of SNP and Indels were evaluated using hap.py benchmarking tools [24] as proposed by Illumina with variants discovered from uncompressed sequencing data as reference. In general, higher impact on both SNP and Indels calling results were observed when the quality values were heavily compressed (Fig. 7 and Supplementary Figure). In addition, compared to STAR, lower F1 scores were obtained when HISAT2 was used as aligner. Noticeably, ScaleQC and LEON brought relatively small impact to the variant calling results despite their high compression ratios. On the other hand, CALQ introduced significantly greater differences to the variant calling results compared to other methods in most cases, in particular for the mouse dataset (SRR1043300) where it only achieved moderate compression gains over lossless compression compared to other tools.

### Computational performance

The run-time and peak memory usage of all lossy compression algorithms on three technical replicates of human chromosome 22 extracted from SRR1050959 were shown in Supplementary Table and Supplementary Table, respectively. Results show that the run-time performance and peak memory consumption vary significantly for different algorithms. Notably, P-Block, R-Block and DSRC completed the compression and decompression operations within 10 seconds while the slowest algorithm (CALQ) took about 30 and 2 minutes respectively to complete the compression and decompression operations for the same file. In terms of memory consumption, Quartz had the highest peak memory consumption (69GB) while Crumble used only around 100MB to perform the compression operation.

We asked if there are any effect on run-time and memory consumption of the downstream RNA-seq data analysis tools if the quality values are lossy compressed. Interestingly, we observed that the differences in run-times and memory consumption resulting from different lossy quality value compression algorithms were actually smaller than those resulting from run-to-run variations when the same algorithm was performed on different technical replicates (Supplementary Figure), indicating that the impact of lossy quality value compression to the run-time and memory consumption of the downstream analysis tools is negligible.

## Discussion

Different RNA-seq tools show different sensitivities to lossy quality compression. For pipelines that do not take base quality values into consideration, e.g., STAR-based expression quantification, analysis results will be reproduced either perfectly after lossy compression or with fluctuations that are irrelevant to quality values if the analysis process is not deterministic. In these scenarios, quality values can be heavily compressed with lossy compression algorithms or can be simply discarded without affecting the analysis results. However, for pipelines that utilize quality values, such as HISAT2 and GATK HC, lossy quality value compression should be used with care in cases where data re-analysis is expected. Short variant discovery is usually not the top-priority application in RNA-seq data analysis. In addition, according to our evaluation, the variations in variant calling results from lossy compressed RNA-seq data among different compression methods were smaller than those among replicates. Hence, we may conclude that quality values can be compressed more aggressively for variant calling applications. However, if re-alignment using a quality value-aware aligner such as HISAT2 is necessary, it is expected that we should only keep the original values intact with lossless compression, or choose a lossy compression algorithm with proper compression ratio to minimize its impact on the alignment results.

When using lossy quality values compression on RNA-seq data, care should also be taken to make sure that the selected tools match the characteristics of the RNA-seq data in hand. For example, Quartz needs a bin directory which was generated according to the human reference genome. Therefore, it does not match the characteristics of sequencing data from other species. As a result, compressing the mouse, rice, and arabidopsis datasets using Quartz actually led to data size increments compared to lossless quality value compression. Some lossy quality compression algorithms were developed primarily for DNA sequencing data analysis. For example, CALQ used an internal statistical model to determine the compression level of quality values from different loci according to their significance. As the model was heavily tuned towards genotyping of DNA sequencing data, CALQ did not perform very well in many RNA-seq tasks in our study. LEON performed well in our benchmarking test as it delivered excellent compression while maintained the accuracy of downstream analysis. However, it does not provide the option to adjust the compression ratio, which makes it inconvenient when the default model does not provide the desired compression level. ScaleQC is a promising tool for RNA-seq data compression as it supports both lossless and lossy quality compression with seamless integration with SAM/CRAM tools, and it provides the flexibility to truncate a bit-stream of lower data rate from that of a higher data rate directly without expensive transcoding. In addition, it was among the compressors that achieved the best trade-off between the compression ratio and impacts to RNA-seq data analysis in our test.

Finally, manufacturers of sequencing platforms have already started to incorporate lossy compression of base quality values into their sequencing hardware platforms to mitigate the storage burden of NGS data. For example, in April 2014, Illumina updated the firmware of HiSeq platforms to enable quality scores binning setting by default. Moreover, currently Illumina Novaseq uses 2-color chemistry, and only consists of four possible quality values which are 2, 12, 23 and 37. The effect of Illumina binning operation on RNA-seq data analysis was studied in this paper using the DSRC implementation, which shows that it has little impact to the downstream analysis of RNA-seq data. However, our study suggests that sequencing data with binned quality values can be further compressed using lossy compression schemes such as LEON or ScaleQC if further file size reduction is desired.

## Conclusion

To overcome the challenge in storage and transmission of NGS data from the increasing production of high-throughput sequencing technologies, compression methods specific for sequencing data have been developed. In these methods, compression of quality values is the most important contributing factor to the overall compression ratio due to the high entropy in quality values. The recent developments in lossy quality value compression methods have brought a new angle to further decrease the data size of NGS data beyond lossless compression. Unfortunately, most of the lossy quality value compressors were developed for and evaluated on DNA sequencing data while their impact on RNA-seq data analysis has yet to be elucidated. In this paper, we provide the first systematic evaluation of existing lossy quality value compression methods on RNA-seq data analysis. Our results reveal that it is viable to further reduce RNA-seq data size without introducing significant adverse effects to downstream analysis, and a suitable choice of the lossy compressor and/or compression ratio parameters heavily depends on the actual pipelines being adopted, and the target applications of the data. Furthermore, given the increasing importance of RNA-seq data analysis in biomedical and clinical studies, it is desirable that established RNA-seq data processing pipelines should be considered in the future development of sequencing data compression algorithms to ensure the applicability of the developed tools on both DNA and RNA sequencing data. Our study thus provides a useful evaluation framework for this development.

## Methods

**Fig. 8:**
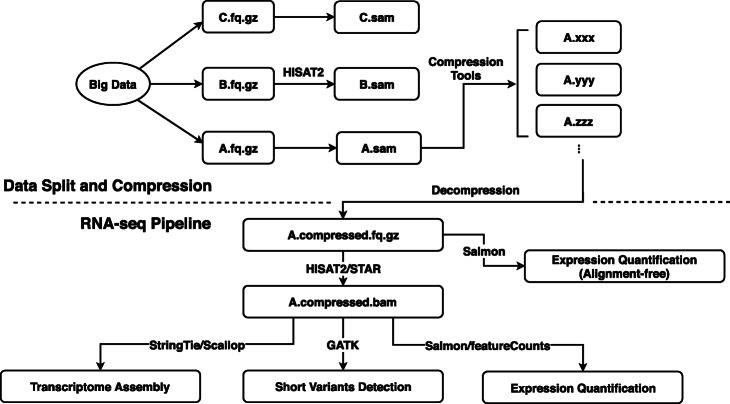
Flowchart of the evaluation pipeline

In this study, we compressed the base quality values in the raw RNA-seq data using different lossy compression tools. The compressed quality data were then decompressed and merged with the other fields of the original RNA-seq data to produce either FASTQ or BAM files for re-analyzing with different pipelines (Fig. 8). The analysis results were compared to those obtained from original data with uncompressed quality values to evaluate the influence of lossy quality value compression on downstream analysis. In our tests, we further divided each evaluation dataset into three parts of same size as technical replicates, with the data amount of each part comparable to that used in conventional RNA-seq studies. The deviation between results from different technical replicates was then used as a baseline to benchmark the significance of the impacts brought by lossy quality value compression to the final analysis results.

### Data and reference genomes

We enrolled four datasets of four different species and platforms in our study (Supplementary Table). Briefly, SRR10509596 was sequenced by HiSeq2000 for the HEK293T human cell line, available from NCBI SRA (https://trace.ncbi.nlm.nih.gov/Traces/sra/?run=SRR10509596), with a total of 35.1 billion bases. We used human hg38 as the reference genome for this dataset. Both the genome sequences and annotation files were download from Ensembl Release 90. SRR10433000 is extracted from mouse endometrial epithelial cells and sequenced using NovaSeq 6000 [25], with a total of 37.2 billion bases. Mouse reference genome mm10 was used as reference for this dataset. The genome sequences and annotation files were also downloaded from Ensembl Release 90. SRR8499098 is HiSeq2500 transcriptome data sequenced from panicle of KitaakeX which is a variety of the *Oryza sativa japonica* (rice) group, and contains a total of 32.5 billion bases [26]. Nipponbare reference genome IRGSP-1.0 was used as reference for this dataset, and both the genome sequences and annotation files were acquired from Ensembl Plants Release 45. SRX4122949 is a combination of two SRA runs SRR7216027 and SRR7216027, with totally 32 billion bases [27]. This dataset contains transcriptome data sequenced from leaf samples of *Arabidopsis thaliana* (Columbia accession) using HiSeq X Ten. Reference genome TAIR10 was selected, and both the genome sequences and annotation files were downloaded from Ensembl Plants Release 45.

Quality control (QC) for all datasets was performed using fastp (v0.19.6) [28] before downstream analysis with parameters “-5 -3 -r -M 20 -q 20”. In addition, “-l 100” was used for the human (SRR10509596), rice (SRR8499098) and arabidopsis (SRX4122949) datasets to set the minimum read length to 100 after trimming. For the mouse dataset (SRR10433000), “-l 80” was used instead since the read length of original data is only 100. Quality information of the original reads and reads after QC is listed in Supplementary Table.

Finally, reads in each dataset were divided equally into three parts (A, B and C) by round-robin before further analysis.

### Data compression

We included eight different lossy quality value compression algorithms (LEON [29], DSRC [30], Quartz [31], CALQ [8], P-block and R-block [6], Crumble [9] and ScaleQC [32]) in our evaluation. Briefly, for compression tools that accept raw genomic sequencing files in FASTQ format such as LEON, DSRC and Quartz, the original FASTQ files were used as input. Otherwise, for tools that accept aligned reads in SAM format, including CALQ, P-block, R-block, Crumble and ScaleQC, the original reads were aligned to their respective reference genome using HISAT2 [19] before compression. Some algorithms support different compression levels through adjusting the corresponding coding parameters. For these algorithms, compression results from the suggested set of coding parameters in the original papers were included in our evaluation.

The output compressed files from these compression tools need to be processed differently to obtain the bit-stream sizes of the compressed quality values. Crumble outputs BAM files which can be further compressed into.cram files using CRAM (by “samtools view”) as suggested in the original paper [9]. ScaleQC also provides an output file that is compatible with CRAM. Quality binning algorithms such as DSRC directly output modified quality values with reduced entropy, which can be further compressed into.cram files. For these tools, the bit-stream size of compressed quality values can be obtained using cram_size available from the CRAM software package. Quartz outputs.fastq files with replaced base quality values. As recommended by the author [31], we used bzip2 with default parameters to further compress the.qual files into.qual.bz2 files. Other tools output a bit-stream file containing only the compressed quality values in their own formats, e.g., CALQ outputs.cq files while P-block and R-block output.cqual files. For these tools, the bit-stream size of compressed quality values is the size of the compressed file in bytes which can be obtained by “wc -c file”. For LEON, the compressed quality values are embedded with other data fields in its own file format and are not accessible. Therefore, we retrieved its quality compression ratio from the log file after compression. Finally, the bit-stream sizes were converted to bits-per-quality (BPQ) values which denote the average number of bits used to encode a base quality value in the compressed bit-stream.

After compression and decompression, in case the decompressed quality values were stored with aligned reads in SAM format, the reads were converted back to FASTQ file format using “samtools fastq” for further processing.

### RNA-seq data analysis

We used the RNA-seq data processing tools encapsulated in the RNA-cocktail toolkits docker image (v0.3.1) [33] in our experiments with default parameters from the accompany RNA-cocktail scripts unless specified otherwise. All the processing pipelines were organized using Nextflow [34]. First, reads were aligned to corresponding reference genomes using either STAR (v2.6.1b) or HISAT2 (v2.1.0). We added “–quantMode TranscriptomeSAM” to the options of STAR so that transcript alignment results can be exported simultaneously with genome mapping. HISAT2 was called through the “run_rnacocktail.py align” command from the RNA-cocktail toolkits.

Subsequently, we used featureCounts (v2.0.0) [35] for alignment-based quantification, and StringTie (v2.0.4) [20] and Scallop (v0.10.4) [36] to detect the transcriptome isoforms base on the alignment results from HISAT2 and STAR. SNPs and Indels were detected through the “run_rnacocktail.py variant” command from the RNA-cocktail toolkit with recommended parameters, which encapsulates the GATK’s Best Practice pipeline [16] on detecting RNA-seq short variants using GATK Haplotype Caller (HC) (v4.1.4.0). For SRR10509596, the human cell line dataset, we used dbSNP v151 as input to the base recalibration process. For the other three datasets, the base recalibration process was skipped.

### Run-time and memory consumption evaluation

To evaluate the processing time and memory expense of different lossy quality value compression algorithms and downstream analysis tools on RNA-seq data with lossy-compressed quality values, we used reads mapped to chromosome 22 of hg38 reference genome extracted from SRR1050959 (the human cell line dataset) as input data (Supplementary Table). All compression tools were tested in a docker container with the number of CPU threads limited to four. Memory consumptions of compression tools were collected using the top command of the Linux OS every 1 second and the peak memory consumptions were recorded for each tool. CPU run-times were also collected from log files produced by top. For RNA-seq data analysis tools, we collected the CPU run-time and peak memory consumption directly from Nextflow with the number of CPU threads limited to six for each container.

All the software tools were run on a single server with dual Intel Xeon E5 2696 v4 (2.2GHz, 88 threads in total), 512 GB DDR4 RAM, quad stripped raid 10 hard disk with 8TB available capacity in total running Ubuntu 16.04.

## Supplementary information

**Additional file 1** Supplementary Data for “Performance evaluation of lossy quality compression algorithms for RNA-seq data”. Supplementary information on software tools, datasets, and additional results in Tables and Figures.

## Abbreviations

NGS: Next Generation Sequencing
DNA: Deoxyribonucleic Acid
RNA: Ribonucleic Acid
QC: Quality Control
BPQ: Bits-per-quality
TP: True Positive
FP: False Positive
FN: False Negative
SNP: Single Nucleotide Polymorphisms
Indel: Insertion and deletion
WGS: Whole Genome Sequencing
WES: Whole Exome Sequencing
DE: Differentially Expressed
MA: Mean-Averaging
