# Supplementary material for: Performance evaluation of lossy quality compression algorithms for RNA-seq data
Source: BMC Bioinformatics. 2020 Jul 20;21:321. doi: 10.1186/s12859-020-03658-4 (PMC7372835; doi:10.1186/s12859-020-03658-4)
Supplement: Supplementary file 1 — Additional file 1 Supplementary Data for “Performance evaluation of lossy quality compression algorithms for RNA-seq data”. Supplementary information on software tools, datasets, and additional results in Tables and Figures. [file 12859_2020_3658_MOESM1_ESM.pdf]

Supplementary Data for “Performance evaluation of lossy quality compression algorithms for RNA-seq data”

Rongshan Yu, Wenxian Yang and Shun Wang

Software Tools

Compresssion tools

- CALQ [1], version 1.0.0, 194fdea (revised version release for Bioinformatics), built from source, (`https://github.com/voges/calq`)
- P-block, R-block [2], da36a12, built from source, (`https://github.com/rcanovas/libCSAM.git`)
- LEON [3], version 1.0.0, executables downloaded from (`http://gatb.inria.fr/software/leon/`)
- Crumble [4], version 0.8.3, 3fbaaaa, built from source, (`https://github.com/jkbonfield/crumble.git`)
- Quartz [5], version 0.2.2, e843195, built from source, (`https://github.com/yunwilliamyu/quartz.git`)
- DSRC [6] (implements IlluminaBinning (`http://res.illumina.com/documents/products/whitepapers/whitepaper_datacompression.pdf`), version 2.00 RC @ 28.03.2014, executables downloaded from (`http://sun.aei.polsl.pl/REFRESH/index.php?page=projects&project=dsrc&subpage=download`)
- ScaleQC [7], version 1.0, built from source, (`https://github.com/xmuyulab/samtools`) and (`https://github.com/xmuyulab/htslib`)

Auxiliary tools

- fastp v0.19.6 [8], (`https://github.com/OpenGene/fastp`)
- STAR v2.6.1b [9], (`https://github.com/alexdobin/STAR`)
- Salmon v0.14.1 [10], (`https://combine-lab.github.io/salmon`)
- SAMtools v1.9 [11], (`https://github.com/samtools/samtools.git`)
- Scallop v0.10.4 [12], (`https://github.com/Kingsford-Group/scallop`)
- GffCompare v0.11.6 [13], (`https://ccb.jhu.edu/software/stringtie/`)`gffcompare.shtml`
- RNACocktail v0.3.1 [14], docker image, (`https://bioinform.github.io/rnacocktail/`), within this image following tools are used:
  - HISAT2 v2.1.0 [15]
  - StringTie v2.0.4 [16]
  - featureCounts v2.0.0 [17]
  - GATK v4.1.4.0 [18]
  - Picard v2.19.0 [18]

Tables and Figures

Table 1: The three datasets for evaluation. Q20 denotes for Phred quality score 20, and "Q20 Bases" refers to number of bases with error probability lower than 0.01. "Q30 Bases" refers to number of bases with error probability lower than 0.001.

		SRR10433000	SRR10509596	SRR8499098	SRX4122949
Species		Mouse	Human	Rice	Arabidopsis
Platform		Novaseq 6000	HiSeq 2000	HiSeq 2500	HiSeq X Ten
Original Reads	Total Reads	372268692	278293552	215361372	213527208
	Total Bases	37226869200	35064987552	32519567172	32029081200
	Q20 Bases	36046681463	33956276012	31232376680	30873969499
	Q30 Bases	34462204755	32818496308	30196154235	29351497969
	Q20 Rates	0.968297	0.968381	0.960418	0.963936
	Q30 Rates	0.925735	0.935933	0.928553	0.916401
Reads after QC	Total Reads	321619968	233179036	186974710	172000696
	Total Bases	31932259843	29038245509	27486760002	25177014623
	Q20 Bases	31488169302	28806704744	27203597468	25005697885
	Q30 Bases	30467694161	28398848396	26734072634	24488163936
	Q20 Rates	0.986093	0.992026	0.989698	0.993196
	Q30 Rates	0.954135	0.977981	0.972616	0.97264

Table 2: Detailed information of reads mapped to human chromosome 22 extracted from three technical replicates (A, B and C) of SRR1050959.

	Total Reads	Total Bases	Q20 Bases	Q20 Rates	Q30 Bases	Q30 Rates
A	1949954	242992130	241023443	0.991898	237487683	0.977347
B	1945310	242421588	240457123	0.991896	236930487	0.977349
C	1946038	242509536	240547771	0.991911	237020341	0.977365

Table 3: Run-times of the lossy compression algorithms. Note that as Quartz and crumble only perform quality value replacement, only compression run-times were recorded for these two algorithms.

Method	Param	A		B		C	
		Comp	Decomp	Comp	Decomp	Comp	Decomp
CALQ	Defalut	30m10s	2m18s	29m29s	2m18s	28m22s	2m16s
DSRC	Defalut	6s	8s	9s	7s	6s	9s
LEON	Default	23s	8s	23s	8s	23s	11s
Quartz	Defalut	6m42s	NA	2m22s	NA	2m17s	NA
Crumble	-1	7m33s	NA	7m20s	NA	7m2s	NA
	-5	5m17s	NA	5m2s	NA	4m59s	NA
	-7	4m58s	NA	4m47s	NA	4m44s	NA
	-9	4m53s	NA	4m51s	NA	4m37s	NA
P-Block	l = 1	8s	2s	8s	1s	6s	1s
	l = 4	6s	1s	5s	1s	6s	2s
	l = 8	5s	<1s	5s	<1s	6s	<1s
	l = 32	6s	<1s	5s	<1s	4s	<1s
R-Block	l = 1.05	8s	1s	8s	1s	7s	2s
	l = 7.4	6s	<1s	5s	<1s	5s	2s
	l = 25	5s	<1s	5s	2s	5s	<1s
	l = 30	5s	<1s	5s	<1s	5s	<1s
ScaleQC	p = 0.1	2m57s	1m44s	2m46s	1m54s	2m44s	1m39s
	p = 0.25	2m33s	1m36s	2m28s	2m2s	2m31s	1m40s
	p = 0.5	2m24s	1m42s	2m31s	2m	2m19s	1m41s
	p = 1	2m18s	1m41s	2m30s	1m59s	2m19s	1m43s
	p = 8	2m16s	1m41s	2m33s	1m52s	2m27s	1m43s

Table 4: Comparison of peak memory usage of lossy compression algorithms on RNA-seq data of human chromosome 22. Note that as Quartz and crumble only perform quality value replacement, only peak memory consumptions of compression operation were recorded for these two algorithms.

Method	Param	A		B		C	
		Comp	Decomp	Comp	Decomp	Comp	Decomp
CALQ	Defalut	5.032G	240.4M	4.325G	206.1M	3.421G	236.2M
DSRC	Defalut	1.967G	738.1M	1.651G	755.2M	2.002G	771.7M
LEON	Default	1.585G	961.1M	1.514G	956.7M	1.532G	959.8M
Quartz	Defalut	69.632G	NA	69.632G	NA	69.632G	NA
Crumble	-1	116.7M	NA	101.5M	NA	129M	NA
	-5	121.9M	NA	101.5M	NA	131.4M	NA
	-7	119.7M	NA	97.4M	NA	126.1M	NA
	-9	117.2M	NA	97.2M	NA	123.6M	NA
P-Block	l = 1	414.7M	231M	414.7M	266.5M	414.6M	200M
	l = 4	26M	262.8M	25.2M	257M	33.4M	229.1M
	l = 8	15.4M	117.2M	108M	172.2M	336M	393M
	l = 32	8.83M	78.2M	9.1M	188.1M	8.8M	94.3M
R-Block	l = 1.05	363.5M	247.5M	363.5M	247M	363.5M	260.7M
	l = 7.4	170.3M	211.4M	25.2M	232.2M	26.9M	247.1M
	l = 25	16.7M	87.5M	16.8M	96.2M	16.9M	123.9M
	l = 30	17.1M	147M	286.9M	234.3M	83.3M	178.9M
ScaleQC	p = 0.1	21.504G	19.456G	32.768G	29.696G	21.504G	19.456G
	p = 0.25	20.48G	22.528G	32.768G	29.696G	21.505G	19.456G
	p = 0.5	20.48G	23.552G	31.744G	29.696G	21.506G	19.456G
	p = 1	21.504G	22.528G	31.744G	29.696G	21.507G	19.456G
	p = 8	20.48G	22.528G	31.744G	29.696G	21.510G	19.456G

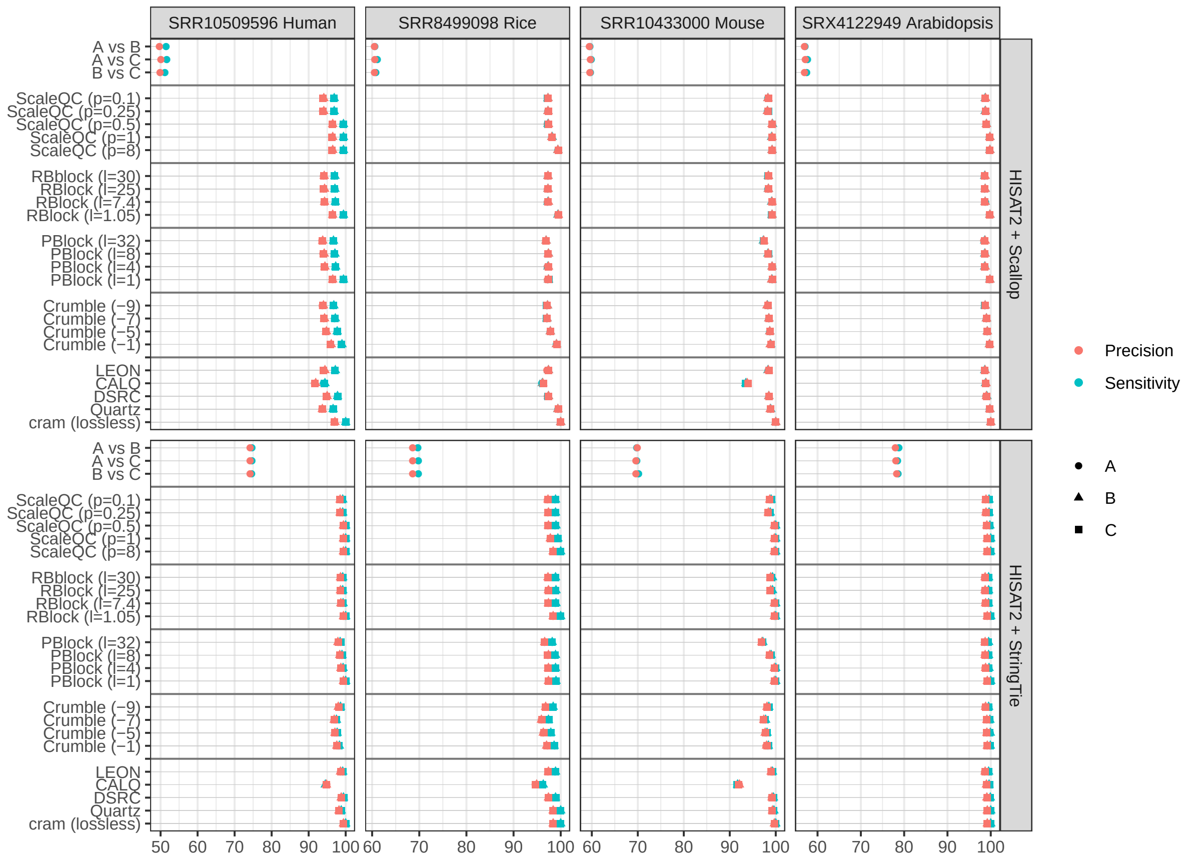

Figure 1: Alignment-based transcriptome assembly comparison. Precision and sensitivity values were computed using GffCompare. Top three lines in each frame represent comparisons of three replicates within each dataset, these comparisons are of assemblies from sequencing data with original quality values. Transcriptomes from reads with lossy-compressed qualities of each replicate were compared with assemblies from uncompressed sequences, precision and sensitivity values were displayed following top three lines representing comparisons of different uncompressed replicates.

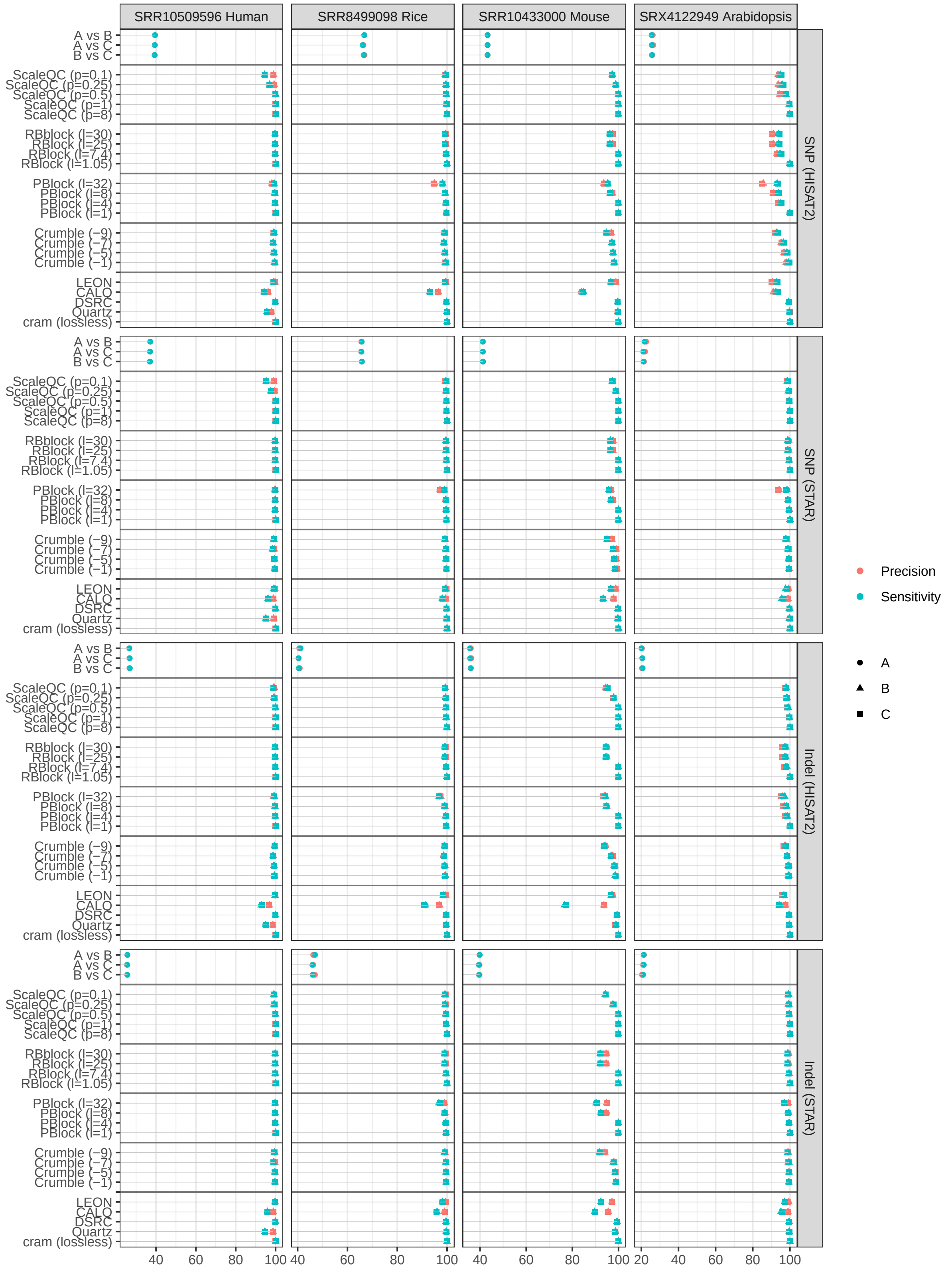

Figure 2: Short variants detection comparison results, precision and sensitivity values were computed using hap.py by Illumina. SNPs and Indels of three replicates detected from alignments with original quality values were compared with each other, and presidios and sensitivity values were shown at top three lines in each frame. Then short variants from compressed reads were compared with variants from uncompressed reads of same replicate, and comparison results were shown in following lines.

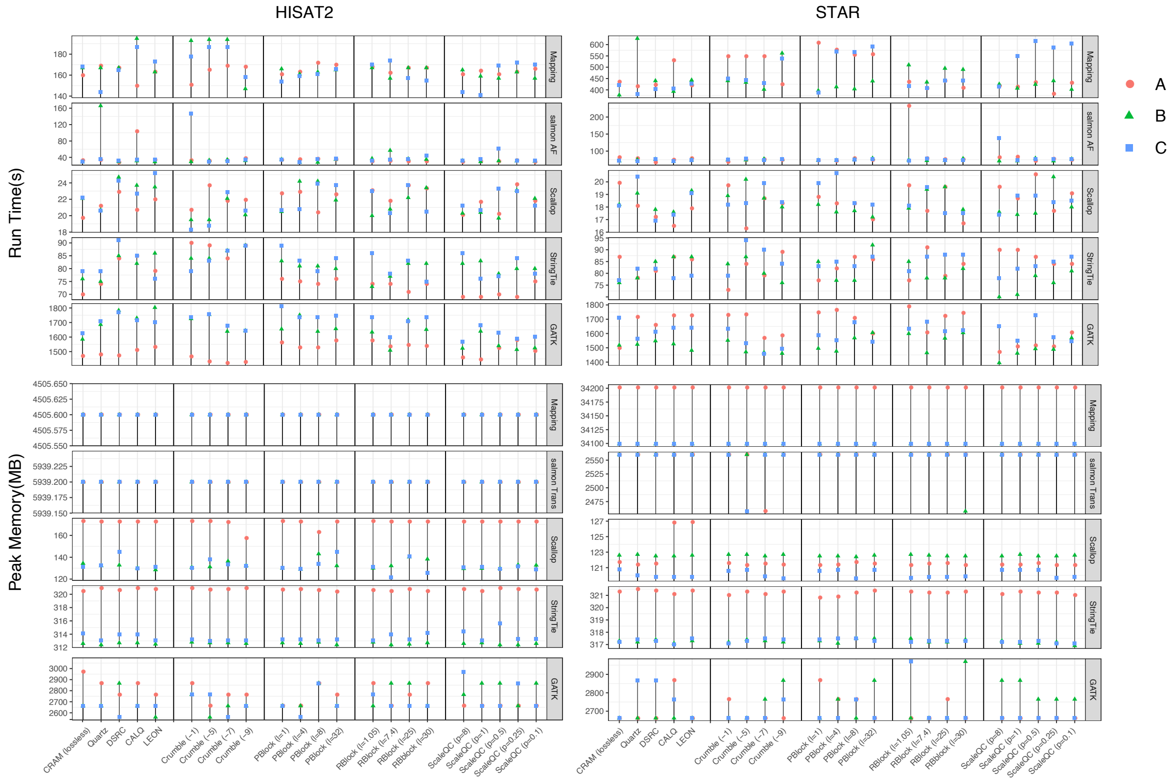

Figure 3: Run-time and peak memory consumption of the RNA-seq data analysis pipelines on RNA-seq data of human chromosome 22 with lossy compressed quality values from different algorithms.

References

[1] Jan Voges, Jörn Ostermann, and Mikel Hernaez. CALQ: compression of quality values of aligned sequencing data. *Bioinformatics*, 34(10):1650–1658, may 2018.

[2] Rodrigo Cánovas, Alistair Moffat, and Andrew Turpin. Lossy compression of quality scores in genomic data. *Bioinformatics (Oxford, England)*, 30(15):2130–6, aug 2014.

[3] Gaëtan Benoit, Claire Lemaitre, Dominique Lavenier, Erwan Drezen, Thibault Dayris, Raluca Uricaru, and Guillaume Rizk. Reference-free compression of high throughput sequencing data with a probabilistic de Bruijn graph. *BMC Bioinformatics*, 16(1):1–14, 2015.

[4] James K. Bonfield, Shane A. McCarthy, and Richard Durbin. Crumble: Reference free lossy compression of sequence quality values. *Bioinformatics*, 35(2):337–339, 2019.

[5] Y William Yu, Deniz Yorukoglu, Jian Peng, and Bonnie Berger. Quality score compression improves genotyping accuracy. *Nature Biotechnology*, 33(3):240–243, 2015.

[6] Lukasz Roguski and Sebastian Deorowicz. DSRC 2–Industry-oriented compression of FASTQ files. *Bioinformatics (Oxford, England)*, 30(15):2213–5, aug 2014.

[7] Rongshan Yu and Wenxian Yang. ScaleQC: A Scalable Lossy to Lossless Solution for NGS Data Compression. *Bioinformatics*, 05 2020. btaa543.

[8] Shifu Chen, Yanqing Zhou, Yaru Chen, and Jia Gu. fastp: an ultra-fast all-in-one FASTQ preprocessor. *Bioinformatics (Oxford, England)*, 34(17):i884–i890, sep 2018.

[9] Alexander Dobin, Carrie A Davis, Felix Schlesinger, Jorg Drenkow, Chris Zaleski, Sonali Jha, Philippe Batut, Mark Chaisson, and Thomas R Gingeras. STAR: ultrafast universal RNA-seq aligner. *Bioinformatics*, 29(1):15–21, jan 2013.

[10] Rob Patro, Geet Duggal, Michael I. Love, Rafael A. Irizarry, and Carl Kingsford. Salmon provides fast and bias-aware quantification of transcript expression. *Nature methods*, 14(4):417–419, apr 2017.

[11] Heng Li, Bob Handsaker, Alec Wysoker, Tim Fennell, Jue Ruan, Nils Homer, Gabor Marth, Goncalo Abecasis, and Richard Durbin. The sequence alignment/map format and SAMtools. *Bioinformatics*, 25(16):2078–2079, aug 2009.

[12] Mingfu Shao and Carl Kingsford. Accurate assembly of transcripts through phase-preserving graph decomposition. *Nature Biotechnology*, 35(12):1167–1169, 2017.

[13] Geo Pertea and Mihaela Pertea. GFF Utilities: GffRead and GffCompare. *F1000Research*, 9:304, apr 2020.

[14] Sayed Mohammad Ebrahim Sahraeian, Marghoob Mohiyuddin, Robert Sebra, Hagen Tilgner, Pegah T. Afshar, Kin Fai Au, Narges Bani Asadi, Mark B. Gerstein, Wing Hung Wong, Michael P. Snyder, Eric Schadt, and Hugo Y.K. Lam. Gaining comprehensive biological insight into the transcriptome by performing a broad-spectrum RNA-seq analysis. *Nature Communications*, 8(1):59, dec 2017.

[15] Daehwan Kim, Ben Langmead, and Steven L Salzberg. HISAT: a fast spliced aligner with low memory requirements. *Nature Methods*, 12:357, mar 2015.

[16] Mihaela Pertea, Geo M Pertea, Corina M Antonescu, Tsung-Cheng Chang, Joshua T Mendell, and Steven L Salzberg. StringTie enables improved reconstruction of a transcriptome from RNA-seq reads. *Nature Biotechnology*, 33(3):290–295, mar 2015.

[17] Yang Liao, Gordon K Smyth, and Wei Shi. featureCounts: an efficient general purpose program for assigning sequence reads to genomic features. *Bioinformatics*, 30(7):923–30, apr 2014.

[18] Aaron McKenna, Matthew Hanna, Eric Banks, Andrey Sivachenko, Kristian Cibulskis, Andrew Kernytsky, Kiran Garimella, David Altshuler, Stacey Gabriel, Mark Daly, and Mark A DePristo. The Genome Analysis Toolkit: a MapReduce framework for analyzing next-generation DNA sequencing data. *Genome research*, 20(9):1297–303, sep 2010.
